# Current treatment landscape of acromegaly

**DOI:** 10.1210/clinem/dgag152

**Published:** 2026-04-08

**Authors:** Frederic Castinetti, Adriana G Ioachimescu

**Affiliations:** Department of Endocrinology, Aix Marseille University, Marseille Medical Genetics, INSERM U1251 and Assistance Publique Hopitaux de Marseille, La Conception Hospital, French Reference Center for Rare Pituitary Diseases HYPO, Marseille 13005, France; Division of Endocrinology and Molecular Medicine, Department of Medicine and Neurosurgery, Medical College of Wisconsin, Milwaukee, WI 53226, USA

**Keywords:** somatostatin receptor ligands, transsphenoidal surgery, paltusotine, radiotherapy, pasireotide, pegvisomant

## Abstract

Treatment of acromegaly includes surgery followed by chronic medical therapy for persistent growth hormone (GH) excess, and, in some patients, radiation. Treatment is aimed at biochemical normalization, which improves survival and comorbidities. However, many patients experience lifelong burden related to persistent acromegaly manifestations and adverse treatment effects. Long-acting somatostatin receptor ligand (SRL) therapy with octreotide or lanreotide has been the cornerstone of management. Pasireotide long-acting release, a somatostatin receptor multiligand, achieves more favorable biochemical control rates but is associated with an increased risk of hyperglycemia. Pegvisomant, a GH receptor antagonist, can be used as monotherapy or in combination with SRLs. The spectrum of medical therapy has expanded with the advent of oral octreotide capsules; the oral selective somatostatin receptor subtype 2 agonist, paltusotine; and monthly self-administered subcutaneous octreotide. This review outlines the updates to current acromegaly treatment options and their impact on patient outcomes.

Since the discovery of injectable somatostatin receptor ligands (SRLs) over 40 years ago, there have been major advances in the medical treatment of acromegaly (Table 1). These include depot and oral SRLs, growth hormone (GH) receptor antagonists, and an oral nonpeptide somatostatin receptor subtype (SSTR) 2 agonist (1). However, transsphenoidal surgery remains the primary treatment and results in remission for many patients at pituitary centers of excellence. Although radiation techniques are considered valuable therapeutic options, their role has been debated over recent years. More focused radiation techniques, such as those using stereotaxy, might redefine their role when long-term safety study results become available. Considering the advances in surgery and medical options, most patients with acromegaly can now achieve biochemical control. However, patients experience lifelong disease burden, including comorbidities that may lead to irreversible sequelae, as well as the adverse events (AEs) associated with treatments. Here, we review the current acromegaly treatment landscape and identify unmet medical needs to improve patient outcomes.

**Table 1:** Medical treatment landscape in acromegaly

Class	Name	Mechanism of action	administration	Biochemical control	Adenoma shrinkage effect	Main side effects
Somatostatin receptor subtype 2 ligand	Octreotide (Sandostatin LAR^®^, Novartis Pharmaceuticals Corporation)	SSTR2 ligand	IM every 4 weeks	30-60%	Yes*^a^*	Gastrointestinal, biliary
	Lanreotide (Somatuline Depot^®^, Ipsen Pharma S.A.S.)	SSTR2 ligand	Deep SC injection every 4-8 weeks	30%-60%	Yes*^a^*	Gastrointestinal, biliary
	Octreotide (Mycapssa^®^, Chiesi USA, Inc.)	SSTR2 ligand	Oral twice daily	58% previously SRL-controlled patients	Insufficient data	Gastrointestinal, biliary
	Octreotide*^b^* (CAM2029, Oczyesa^®^, Camurus AB)	SSTR2 ligand	SC every 4 weeks	75% previously SRL-controlled patients	Insufficient data	Gastrointestinal, biliary
Nonpeptide somatostatin receptor agonist	Paltusotine (Palsonify™, Crinetics Pharmaceuticals, Inc.)	SSTR2 agonist	Oral once daily	83% previously SRL-controlled patients	Insufficient data	Gastrointestinal, biliary
Somatostatin receptor multiligand	Pasireotide (Signifor^®^ LAR, Recordati Rare Diseases Inc.)	SSTR2 and SSTR5 ligand	IM every 4 weeks	50%*^c^*	Yes	Gastrointestinal, biliary, hyperglycemia, and diabetes
GH receptor antagonist	Pegvisomant (Somavert^®^, Pfizer Inc.)	GH receptor antagonist	SC injection daily	70%	No	Increased transaminase (rare) lipodystrophy
Dopamine receptor agonist*^d^*	Cabergoline (Dostinex^®^, Pfizer Inc.)	D2R agonist	Oral twice a week	30%	Insufficient data	Gastrointestinal, orthostatic hypotension, dizziness

Abbreviations: D2R, dopamine type 2 receptor; GH, growth hormone; IM, intramuscular; LAR, long-acting release; SC, subcutaneous; SRL, somatostatin receptor ligand; SSTR2, somatostatin receptor subtype 2; SSTR5, somatostatin receptor subtype 5.

^*a*^Adenoma reduction rates higher with primary treatment.

^*b*^Not approved in the United States.

^*c*^Pasireotide LAR antisecretory efficacy was demonstrated in 20% of patients resistant to octreotide LAR or lanreotide depot.

^*d*^Off-label use for acromegaly.

## Pituitary surgery

GH-secreting pituitary adenomas (PAs) are usually macroadenomas with a high propensity for cavernous sinus invasion. Transsphenoidal resection of GH-secreting PAs by an experienced neurosurgeon is currently recommended as first-line therapy (1). Surgery achieves rapid sellar decompression, reduction in adenoma size, and attenuated GH levels, along with improved symptoms. At pituitary centers of excellence, permanent endocrine complications such as de novo anterior hypopituitarism or arginine vasopressin deficiency are rare after removal of GH-secreting PAs (2).

Primary medical treatment can be considered in patients who refuse surgery or those with unacceptable surgical risk, uncontrolled comorbidities such as sleep apnea and heart failure, lack of access to specialized neurosurgeons, or largely unresectable tumors located primarily in the cavernous sinuses (3).

Surgical remission is defined by achieving age-appropriate normalized insulin-like growth factor 1 (IGF-I) levels measured after 3 months and confirmed by a nadir GH of <0.4 ng/mL during an oral glucose tolerance test (4). Notably, discordant postoperative IGF-I and GH results occur in >25% of patients. Patients with normal IGF-I but insufficient GH suppression can be carefully monitored without treatment, while considering the possibility of mild ongoing disease activity (5) and other factors that influence the GH nadir, such as sex, estrogen use, and body mass index (BMI). Conversely, when postoperative GH levels are low, but IGF-I is mildly elevated short-term after surgery, biochemical evaluation should be repeated. IGF-I delay to normalization is explained by the long half-life of IGF-I and IGF-binding proteins (6).

Surgical remission rates of 32% to 85% are reported in individual studies, depending on patient population and criteria used to define remission, as well as adenoma size. In meta-analyses, remission occurred in 78% of patients with microadenomas, 53% of patients with macroadenomas, and 69% of patients with noninvasive PA (vs 29% remission for invasive PAs) (7). Surgical experience in pituitary resections is associated with more favorable biochemical outcomes, whereas no significant differences were noted by studies comparing endoscopic with microscopic approaches (8, 9). Factors determining more favorable remission rates include dedicated pituitary-focused neurosurgeons, concordantly with improved resolution of preoperative magnetic resonance imaging (MRI) and advances in surgical techniques (2, 10). A recent study reported that cavernous sinus invasion and infrasellar extension >5 mm were predictive markers of incomplete resection (11). Reoperation of GH-secreting macroadenomas is associated with lower remission rates (27.5%) (12) and may be indicated for patients with large residual adenoma tissue who do not respond to medical therapy.

Early identification of patients who are likely to experience persistent acromegaly postoperatively may shorten the time to biochemical control with medical treatment. Preoperatively, the main predictors of persistent biochemical disease are higher GH levels, larger adenoma size, and Knosp grade 3 to 4 cavernous sinus invasion (9, 13-16). Conversely, low serum GH levels in the first few days postoperatively are strongly associated with long-term surgical remission (17-22). Histological parameters including mitosis index and prolactin co-secretion may predict persistent biochemical activity or adenoma growth. One study showed lower (albeit not statistically significant) surgical remission rates for prolactin co-secreting (18%) compared with pure GH-secreting PA (32%) (23). Another study showed similar remission rates in the 2 groups (53%), but a higher occurrence of postoperative arginine vasopressin deficiency for co-secreting PAs (24).

Biochemical recurrence after initial postoperative remission of up to 18.5% was reported in individual studies, depending on biochemical criteria used (8). In a recent study of 283 patients, 132 achieved postoperative remission, and only a minority (4 patients, 3%) experienced recurrence after a median time of 87 months (25). Recurrence was defined by increased IGF-1 levels; notably, there was no evidence of tumor regrowth radiologically in these cases (25).

In the first year postoperatively, IGF-I measurement is recommended every 3 to 6 months and MRI after ≥3 months. Subsequently, IGF-I should be measured every 6 to 12 months in the first 5 years postoperatively and on an individualized basis thereafter. Imaging should be repeated dependent on biochemical parameters, findings of the initial postoperative MRI, and adenoma histological parameters (1). However, lifelong monitoring and management of acromegaly complications are required.

In summary, surgery by dedicated neurosurgeons at high-volume multidisciplinary centers is the primary treatment for most patients with acromegaly. Preoperative biochemical, radiological, and histological parameters, as well as GH levels in the first week postoperatively can identify patients who require subsequent medical therapy.

## Medical treatments

### Long-acting injectable somatostatin receptor subtype 2 ligands

Octreotide and lanreotide act mainly by binding to SSTR2, which is expressed on the somatotroph adenoma. Long-acting octreotide requires intramuscular injections every 4 weeks (octreotide long-acting release [LAR]), whereas lanreotide autogel (ATG) requires deep subcutaneous injections every 4 to 8 weeks. SRLs are the first-line treatment for patients in whom surgery is contraindicated, those with a low probability of achieving durable surgical remission (eg, somatotroph adenomas with cavernous sinus invasion), or for persistent GH hypersecretion after surgery (26, 27). The control rate obtained with these medications varies from 21% to 43%, depending on whether SRLs are used as primary or adjuvant treatment, dose titration, and maximum dose attained (28-30). Efficacy may be underestimated because the maximum dose is not always delivered. For example, in the US MarketScan database, the mean doses of lanreotide and octreotide were 90 and 20 mg every 28 days, respectively, yet acromegaly was not controlled in all patients (31). Use of a higher dose or a high-frequency regimen achieved control in 25% to 30% of patients (32). However, in the US MarketScan database, this was only reported for a small percentage of patients (5%) (31). Conversely, patients who are biochemically controlled can take advantage of extended dosing intervals, which are effective in >70% of patients (33).

The main predictors of therapeutic response identified in multiple studies include IGF-I levels (34), cytoplasmic SSTR2 expression, the GH granulation pattern, and T2-weighted signal intensity detected on MRI (35) (Table 2). Aryl hydrocarbon receptor interacting-protein (AIP) is possibly implicated in the SSTR2 signaling pathways and response to SRL, with some studies showing lower response in patients with germline *AIP* mutations and those with lower AIP expression within the PA cells (27). No single parameter predicts biochemical response. A recent study in 67 medically naive patients (of whom 37 were responders) proposed a multibiomarker score based on cytoplasmatic SSTR2 expression, residual postoperative adenoma tissue, and the CD68^+^/CD8^+^ immune cell ratio (36). Machine learning models have been developed to predict efficacy as determined by age, sex, pretreatment GH and IGF-I levels, and molecular markers, including GH granularity, adenoma expression of E-cadherin, ghrelin pre-propeptide, intron 1 retained ghrelin variant, D2 dopamine receptor, SSTR5, and phosphatidylethanolamine-binding protein 1 (37-39). The ACROFAST study prospectively compared treatments based on results of the short acute octreotide test results, adenoma T2-MRI signal, and E-cadherin immunostaining and suggested that this approach could help achieve GH hypersecretion control more quickly by improving the initial drug selection (40). However, biomarkers and T2-MRI signal evaluation are not routinely performed in clinical practice. Accordingly, initial therapy with SRLs could be undertaken in most patients.

**Table 2:** Predictors of incomplete biochemical response to long-acting injectable SSTR2 ligand medications (octreotide, lanreotide) in acromegaly

Factors	Associated with incomplete biochemical control	Independent predictor in clinical studies
Demographic		
Age	Young (<40 years)	
Sex	Male	
Anthropometric		
Bodyweight	Higher	Yes
Body mass index	≥30 kg/m^2^	
Comorbidities		
Type 2 diabetes	Present	
Biochemical		
Pretreatment IGF-I levels	Higher	Yes
Pretreatment GH levels	Higher	
Imaging		
T2-weighted signal on MRI	High intensity	Yes
Adenoma size, extension, and cavernous sinus invasion	Larger, with extrasellar growth, higher Knosp grade	
Adenoma histopathology		
SSTR2 expression	Lower IHC score or % positive cells	Yes
Granulation pattern	Sparsely granular	
ki-67 proliferation index	High	
E-cadherin	Negative	

Abbreviations: GH, growth hormone; IGF-I, insulin-like growth factor 1; IHC, immunohistochemistry; MRI, magnetic resonance imaging; SSTR2, somatostatin receptor subtype 2.

SRLs may also be used preoperatively for severe comorbidities that could make intubation more difficult, such as severe pharyngeal swelling, sleep apnea, or severe heart failure. They might also be used for patients with large and invasive macroadenomas by inducing adenoma shrinkage in 50% to 80% of patients, which may make surgery easier by decreasing adenoma size (41-43). Recent studies suggest that SRLs may also modify the tumor microenvironment (44, 45). While theoretically surgical outcomes may thus be improved—especially at centers with non-expert neurosurgeons—this approach has not been uniformly validated (3, 46). Concordant with this, in a meta-analysis on preoperative use of SRLs, the authors reported a greater short-term effect which was not sustained long-term. The specific group of patients with invasive macroadenomas were not evaluated per se (47).

With long-term use, injectable SRLs can contribute to increased disease burden and decreased quality of life (QOL), with potential impact of adherence to treatment. The main AEs are gastrointestinal (48). However, injections themselves are also burdensome. More than 80% of patients complain of injection pain, 50% report bruising (49), and 16% experience a loss of workdays (50). For lanreotide ATG, at-home injections can be administered by the patient or a partner. However, patient preference between health care professionals and partner administration is not uniformly accepted (51). A nurse-led home injection service may increase adherence (52). Timing and convenience of treatment injections, as well as patient independence, might favor the use of lanreotide ATG vs octreotide LAR (53). A recent study focused on disease burden in 146 patients receiving SRLs using the Acromegaly Treatment Satisfaction Questionnaire (Acro-TSQ), a patient-reported outcome measure developed specifically for patients with acromegaly receiving oral or injectable treatment. Acro-TSQ assesses symptom interference, gastrointestinal side-effect interference, injection-site interference, treatment satisfaction, emotional reactions, and treatment convenience. Two-thirds of patients reported experiencing persistent symptoms of acromegaly, with 50% mentioning that these symptoms worsened in the days prior to the next injection and interfered with their daily lives. Interestingly, about 50% were frustrated with their treatment regimens and two-thirds felt less independent (54). A recent study showed that IGF-I levels tended to rise between weeks 1 and 4 after injection, associated with deteriorating QOL scores and fluctuated throughout the 4-week period (55). Extended dosing intervals improved patients' experiences (33).

**Figure 1:**
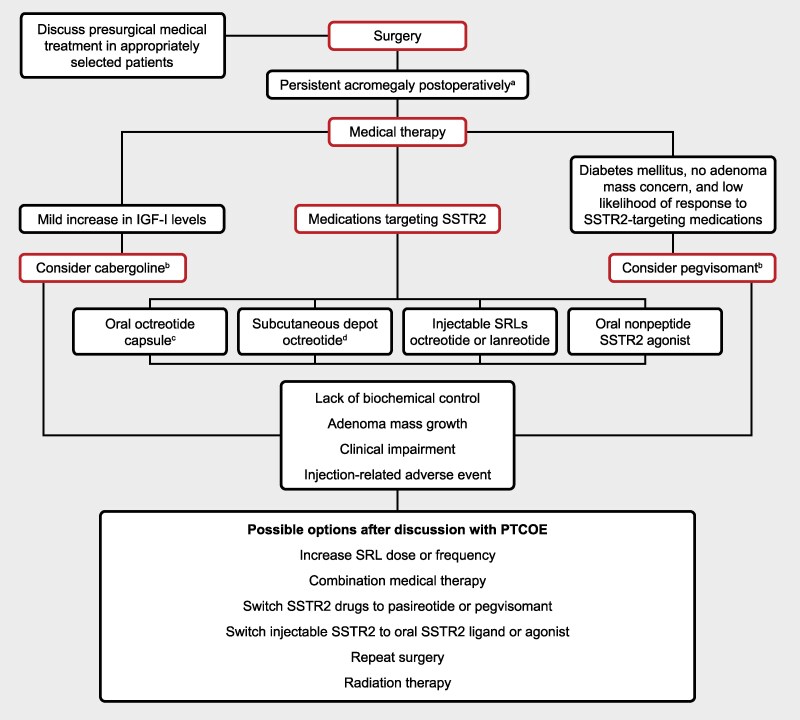
Treatment landscape in acromegaly. ^a^Consider reoperation in selected cases with large surgically-accessible residual adenomas. ^b^Indication as a first-line treatment is not approved in all countries; cabergoline is not FDA-approved for acromegaly. ^c^FDA approved for responders to injectable octreotide LAR and lanreotide depot. ^d^Not approved in all countries. Possible options after discussion with PTCOE: Persistent adenoma growth may raise the concern of an aggressive adenoma. Treatment options should be discussed in multidisciplinary tumor boards to include surgery, radiation techniques, addition of SRLs to pegvisomant monotherapy, or switch from octreotide or lanreotide to pasireotide or combination of pasireotide to pegvisomant. Close MRI monitoring should be performed. Adapted from Melmed et al Consensus on acromegaly therapeutic outcomes: an update. *Nat Rev Endocrinol*. 2025;21(11):718-737 with permission from Springer Nature. Abbreviations: IGF-I, insulin-like growth factor 1; PTCOE, Pituitary Tumor Centers of Excellence; SRL, somatostatin receptor ligand; SSTR2, somatostatin receptor subtype 2.

Although SRLs are considered the primary medical treatment for persistent disease after transsphenoidal surgery (Fig. 1), they are only effective in 40% of unselected patients. SRL injections also may not resolve the clinical disease burden even while achieving biochemical control and may cause inconvenience and injection-related AEs. Thus, there are unmet medical needs for this initial treatment. Additional drugs targeting somatostatin receptors have been approved by the US Food and Drug Administration (FDA) and/or the European Medicines Agency (EMA).

### Oral octreotide capsule

Oral octreotide is an acetate capsule formulation (OOC) with improved absorption due to a Transient Permeability Enhancer^®^, allowing for transient opening of intestinal tight junctions (56). OOC is initiated at a dose of 20 mg twice a day, ≥1 hour before or 2 hours after meals. Dose adjustments in 20-mg increments can be made every 2 weeks based on IGF-I level and symptoms.

OOC was evaluated in 2 main phase 3 clinical trials (57). The CHIASMA OPTIMAL phase 3, double-blind, multicenter study aimed at evaluating efficacy and safety of the compound in 56 patients whose condition was controlled by injectable SRLs. At the end of the trial, 16/28 (58.2%) of patients on OOC vs 4/28 (19.4%) of patients on placebo (*P* = .008) had normal IGF-I. Fifty-five patients (98.2%) experienced ≥1 AE, most of which were gastrointestinal (58, 59).

The MPOWERED open-label, multicenter, randomized study evaluated the noninferiority of OOC compared with SRLs in 92 patients previously controlled by injectable SRLs. The primary endpoint was the proportion of patients with IGF-I levels below the lower limit of normal. After 62 weeks, 91% of patients in the OOC group (*n* = 50/55) and 100% of patients in the SRL group (*n* = 37) maintained biochemical control; the non-inferiority criterion was met. Notably, 71 patients experienced ≥1 AE, primarily gastrointestinal (60). Forty-five patients (75%) completed the extension study, and approximately 90% of them remained controlled (61).

The EMA has granted OOC orphan indication for treatment of acromegaly, and the FDA has approved it for patients who have demonstrated a complete or partial response to injectable SRLs. Its oral administration, similar efficacy to injectable SRLs, and side effect profile provides patients who complain of the burden of injections with another option (62).

### Subcutaneous depot octreotide

A new formulation of depot octreotide using the FluidCrystal technology was recently developed for subcutaneous administration (CAM2029). Results of the ACROINNOVA program showed similar biochemical efficacy of CAM2029 with SRLs and improved acromegaly symptom control and QOL. ACROINNOVA 1, a randomized, double-blind, placebo-controlled phase 3 study, enrolled 72 patients previously controlled on SRLs. At 22 weeks, more patients in the CAM2029 group achieved IGF-I normalization (75%) compared with placebo (38%), along with well-controlled acromegaly symptoms and higher QOL and treatment satisfaction scores compared with pretreatment and placebo (63). ACROINNOVA 2, an open-label phase 3 study (52 weeks), showed persistent biochemical effects of CAM2029 and no new AEs. Fifteen percent of patients previously uncontrolled on octreotide LAR or lanreotide ATG achieved normal IGF-I. New injection-site AEs were mild-to-moderate and decreased over the study duration, leading to treatment discontinuation in 1.5% of patients (64).

Subcutaneous depot octreotide was approved by the EMA for adult patients with acromegaly who have responded to and tolerated treatment with SRLs. The medication is self-administered subcutaneously at a dose of 20 mg (1.0 mL) once a month (±7 days) using a pre-filled autoinjector pen with a hidden, thin needle. It is currently not approved by FDA. Subcutaneous depot octreotide might be an option for patients preferring self-administration of their monthly injectable SRL medication.

### Oral nonpeptide somatostatin receptor agonist: paltusotine

Paltusotine is an oral, once-daily, nonpeptide molecule and potent, SSTR2 agonist (65). It has efficient digestive absorption, allowing for oral delivery, and is taken ≥6 hours after a meal and 1 hour before the next meal (66).

The PATHFNDR-1 phase 3, randomized, double-blind, placebo-controlled study assessed efficacy and safety of paltusotine in patients previously treated with SRLs. Patients were switched from injected SRLs and randomized to receive paltusotine or placebo orally for 36 weeks. The primary endpoint was proportion of patients maintaining IGF-I ≤ 1.0× ULN. By the end of the study, 25 of 30 patients (83.3%) in the paltusotine group and 1 of 28 patients (3.6%) in the placebo group had reached the primary endpoint (*P* < .0001). IGF-I levels remained stable in the paltusotine group, whereas they doubled in the placebo group. At least 1 AE—primarily gastrointestinal—occurred in 80% of patients in the paltusotine group and 100% of patients in the control group. No change in adenoma size was observed (67).

The results of the phase 3 PATHFNDR-2 randomized, placebo-controlled, multicenter study showed that 56% of participants taking paltusotine achieved normal IGF-I levels, compared with 5% of those taking a placebo. Paltusotine was generally well tolerated, with no serious AEs (68).

The FDA approved paltusotine to treat adults with acromegaly who have had an inadequate response to surgery and/or are not eligible for surgery. Paltusotine also received orphan drug designation from the EMA for treating acromegaly. At the end of February 2026, the Committee for Medicinal Products for Human Use recommended authorization of Palsonify, for the treatment of adults with acromegaly. Its single oral administration and similar efficacy to SRLs provides patients who complain of the burden of injections with another option (69). Paltusotine can be initiated at 40 mg daily with possibility to increase (to 60 mg) or decrease (to 20 mg) after IGF-I measurement at 2 to 4 weeks.

### Somatostatin receptor multiligands

Pasireotide is a somatostatin receptor multiligand which, in addition to acting on SSTR2, binds with high affinity to SSTR5.

In a phase 3 head-to-head study in 358 medically naive patients randomized to monthly intramuscular octreotide or pasireotide LAR, 31.3% patients taking pasireotide and 19.2% patients taking octreotide achieved normal IGF-I and GH levels <2.5 ng/mL (70). In a phase 3 trial, 198 uncontrolled patients were randomized to pasireotide LAR (40 or 60 mg) or to their continued treatment with octreotide or lanreotide (active control) (PAOLA). At 24 weeks, 20% of patients taking pasireotide met the IGF-I normalization goal along with GH levels below 2.5 ng/mL, compared with no patients in the active control group (71). PAOLA extension trial indicated that 37% of patients taking pasireotide maintained control after 5 years (72). In 61 patients taking SRL (octreotide or lanreotide) and pegvisomant combination, switching to pasireotide controlled IGF-I level in most patients with a pegvisomant-sparing effect of 66% after 25 weeks; however, the prevalence of diabetes increased from 33% at baseline to 69% during the study (73).

Pasireotide significantly reduced adenoma size in 37.7% of treated patients (74). In the PAOLA study, tumor shrinkage >25% was noted in 18.5% of patients previously uncontrolled by octreotide or lanreotide (71). Real-life studies confirmed clinical trial findings on adenoma shrinkage (75), which can occur in the absence of complete biochemical control (76).

Factors predicting pasireotide response include baseline IGF-I level (75), SSTR2 and SSTR5 expression, and MRI signal intensity on T2 images (77).

Similar to octreotide and lanreotide, pasireotide can cause gastrointestinal, biliary, nutritional (vitamin B12 deficiency), and injection-related AEs. Additionally, pasireotide is associated with an increased risk of hyperglycemia and QT prolongation. New or worsening hyperglycemia occurs in 65% to 75% of patients with acromegaly treated with pasireotide, with higher likelihood for age >40 years, BMI > 30 kg/m^2^, and preexisting hyperglycemia, hypertension, or dyslipidemia (78). Mean hemoglobin A_1c_ increased by 0.6% to 0.7% in patients treated with pasireotide and did not change in patients treated with octreotide during the head-to-head phase 3 trial (70). Hyperglycemia is due to decreased insulin and incretin secretion. Treatment of pasireotide-induced hyperglycemia includes metformin or incretin-based medications (79). To evaluate the QT interval, electrocardiogram is recommended at baseline, 3 weeks after treatment initiation, and with each medication uptitration. Hypokalemia, hypomagnesemia, and hypothyroidism should be corrected before pasireotide initiation, and concomitant medications that could prolong the QT interval should be avoided.

Long-acting pasireotide administered intramuscularly every 28 days was approved by the FDA for treatment of patients who have had an inadequate response to surgery and/or for whom surgery is not an option. The EMA indication is for adult patients with acromegaly for whom surgery is not an option or has not been curative, and whose disease is inadequately controlled on treatment with another SRL. Pasireotide is initiated at a dose of 40 mg monthly, injected intramuscularly by a health care professional. The dose can be increased after 3 months to 60 mg monthly if acromegaly remains uncontrolled.

The 2020 acromegaly consensus guidelines recommended monotherapy pasireotide for patients not controlled on SRLs and/or pegvisomant. For patients not achieving adequate control on pasireotide monotherapy, combination with pegvisomant can be considered (80), as detailed later.

### GH receptor antagonist

Pegvisomant is a recombinant GH receptor antagonist used as a second- or third-line treatment for patients who have had unsuccessful transsphenoidal surgery and who have partial or complete resistance to SRLs (81, 82). Pegvisomant can be used as monotherapy in patients with complete SRL resistance and with no adenoma mass concerns, or in combination with SRLs, dopamine receptor agonists, or pasireotide (82-85).

While initial studies suggested that pegvisomant could control hypersecretion in 90% of patients, real-world data reported 60% to 70% efficacy. This was attributed to insufficient pegvisomant uptitration in some patients. The main predictive factor of remission is the pretreatment IGF-I level, with age, sex, and BMI inconsistently demonstrated (86-88). In patients treated with pegvisomant, IGF-I is the only available biomarker to determine drug efficacy. ACROSTUDY, a global, multicenter, observational, pharmaco-epidemiological surveillance study in 2077 patients, emphasized the need to achieve normal IGF-I levels to avoid increased mortality due to cardiovascular, cerebral, malignant, and respiratory diseases. Other risk factors for premature mortality included young age, radiotherapy, and duration of acromegaly prior to treatment (87).

For patients with uncontrolled acromegaly taking SRLs, adenoma volume, glucose metabolism, and the patient's injection preference should be considered prior to changing therapy. Pegvisomant has no antitumor effect and should be considered for monotherapy only in patients for whom adenoma growth control is not the primary goal. However, the natural progression of adenoma growth may be a concern. While some aggressive adenomas grow, others may continue to grow when SRLs are switched to pegvisomant (89, 90). The reported rate of increase of 2% to 7% of patients (91, 92), might thus be overestimations.

Pegvisomant monotherapy has a favorable effect on glucose, improving glucose tolerance (93, 94), by blocking GH receptors in the fat tissue and liver (95, 96).

Pegvisomant may also exacerbate the disease burden due to required frequent subcutaneous injections. This may explain why QOL scores only slightly improved during treatment, regardless of the level of IGF-I control achieved (97). These scores are similar to those of patients taking octreotide LAR or lanreotide ATG (98). These studies, however, were based on a limited number of patients, and did not take into account some factors that might also influence quality of life independent from the treatment (previous therapy, patients' expectations, duration of treatment…). As such, formal conclusions are not feasible.

Pegvisomant is typically administered daily via subcutaneous injection, but the total weekly dose can be administered less often (once to several times/week) with similar efficacy (92, 99, 100). Adherence to treatment ranges from 61% to 92% and is lower in older patients and in those without a regular daily injection schedule. Notably, one-third of patients complained of mistakes during drug reconstitution (101). Lipodystrophy at the injection site is observed in <3%. Transiently increased liver enzymes are observed in up to 6%, and are more common when combining pegvisomant and SRLs (91).

Long-term data on the real-world use of pegvisomant demonstrated high efficacy and a favorable tolerance profile. However, since the drug only acts at a peripheral level, it must be combined with SRL in patients with concerns about persistent adenoma growth, as detailed in the next paragraph. Pegvisomant monotherapy has a favorable effect on glucose, improving glucose tolerance (93), by blocking GH receptors in the fat tissue and liver, making it the drug of choice for patients with poorly controlled diabetes (Fig. 1).

### Pegvisomant and SRL combination treatment

Pegvisomant combination with long-term injectable SSTR2 ligands (octreotide LAR or lanreotide depot) has been used for patients with partial resistance to SRLs and tumor mass concern. Initial studies reported IGF-I control in up to 90% of cases (102-104). In the ACROSTUDY, among the 768 patients treated with this combination, 62% achieved control after 4 years of treatment (83). A recent meta-analysis reported IGF-I control in 66% patients (105). These lower rates are probably due to insufficient uptitration of pegvisomant. By combining the 2 treatments, the necessary dose of pegvisomant is usually lower compared with pegvisomant monotherapy. One explanation for the lower dose of competitive GH receptor blocker is the effect of the SRL on GH levels and hepatic GH receptors (106-108). Dose of pegvisomant used in combination therapy positively correlates with pretreatment IGF-I level normalized by age, weight, and height (87).

Combination of SRL with pegvisomant improves QOL (109) and glucose metabolism (110) compared with SRL monotherapy. Tumor size control or shrinkage can be observed in most patients, which is not the case for pegvisomant monotherapy (104). Combination therapy also reduces annual treatment costs without compromising efficacy. Bonert et al demonstrated that a combination of low-dose SRL (lanreotide 60 mg or octreotide 10 mg every 4 weeks) plus weekly pegvisomant (40-160 mg/week) is an effective and cost-effective treatment option for patients with acromegaly, regardless of their response to prior SRL (111). Similar results could be expected with pegvisomant combined with new compounds targeting SSTR2; however, no studies have evaluated this.

Pegvisomant and pasireotide combination has been reported in few studies. Muhammad et al reported on the efficacy and safety of switching from a combination of injectable SRLs and pegvisomant to pasireotide monotherapy or a combination of pasireotide and pegvisomant. Pasireotide monotherapy enabled the withdrawal of pegvisomant in 67% of patients; for the remaining patients, combining pasireotide and pegvisomant resulted in a 66% reduction in pegvisomant dose. AEs of grades 1-2 hyperglycemia were reported in 66% of patients, while grades 3-4 were reported in 23%, indicating that pegvisomant cannot overcome pasireotide-induced glucose abnormalities (73). This combination could also be considered for patients with tumor growth and/or uncontrolled IGF-I levels despite taking pegvisomant and SRLs, with close glucose monitoring (112). Case series suggest the utility of this combination for selected patients with large, invasive adenomas resistant to other medical therapies (113) and propose that baseline GH levels may predict worsening glucose metabolism (114).

### Dopamine receptor agonists

Cabergoline is a dopamine receptor agonist approved for the treatment of hyperprolactinemia. Because GH-secreting PAs express dopamine receptors type 2, cabergoline has been used in acromegaly (off-label).

Based on retrospective studies, the efficacy of cabergoline monotherapy is modest (115, 116). In a prospective study in 24 patients followed for 18 weeks, 11% of patients achieved normal IGF-I levels after titration to 0.5 mg of cabergoline daily (117). In a meta-analysis, patients with baseline IGF-I levels <1.5× above the upper limit of normal had the highest rates of biochemical control, and the mean cabergoline dose in responders was high at 2.5 ± 1.4 mg/week (118).

Cabergoline combination with monthly octreotide LAR or lanreotide ATG normalizes IGF-I levels in 50% of patients based on a meta-analysis. Patients with lower baseline IGF-I levels were more likely to respond (118). Adding pegvisomant 10 mg daily to cabergoline 3.5 mg/week was shown to normalize IGF-I levels in 68% of patients (117). A retrospective study in 14 patients resistant to SRLs who did not achieve biochemical control after pegvisomant monotherapy (10-30 mg/day) found that adding cabergoline (1-3 mg/week) normalized IGF-I levels in 28% and improved IGF-I in 64% of patients (119). In the prospective MPOWERED study, cabergoline (1-3.5 mg/week) was added in 14 patients not controlled on oral octreotide alone; the combination achieved the IGF-I goal in 56% of patients without serious AEs (120). It is, however, important to consider that, due to the low number of patients evaluated, firm conclusions on the benefit of such an association remain debatable.

AEs of cabergoline are like those encountered in patients treated for prolactinoma, although data regarding psychological changes and valvulopathy are limited in acromegaly. Current consensus guidelines indicate cabergoline monotherapy can be used for patients with mild IGF-I elevation after surgery and those with GH- and prolactin-secreting adenomas (1). Use of cabergoline in combination with SRLs and/or pegvisomant can also improve biochemical control in acromegaly.

## Radiotherapy

Radiation techniques are usually considered third-line treatments (ie, after unsuccessful transsphenoidal surgery and poor tolerance or resistance to medical treatments) or for aggressive PAs. Several modalities are available, ranging from stereotactic to conformal while passing by proton beam. While their use has markedly decreased over the last 15 years (121, 122), it is likely that modern, more focused approaches still have a role as stated in recent guidelines (81).

Stereotactic approaches can be delivered in a single session (Gamma Knife radiosurgery) or with fractionation of the total dose (fractionated stereotactic radiotherapy) (123). Stereotaxy uses a headframe to precisely target the tumor remnant: this approach is mainly considered for small, well-defined tumor remnants. For radiosurgery, the remnant should be sufficiently far from the optic chiasm to avoid a risk of optic neuritis. The safety of critical surrounding structures is based on the principle of “precision sparing,” meaning that the high accuracy of the technique should avoid damaging surrounding structures. Antisecretory efficacy reaches 50% to 70% of cases (124, 125). As for all radiation techniques, maximal efficacy is usually observed after 3 to 5 years with a progressive decline in GH levels. An effective antisecretory drug is required while waiting for maximal efficacy to ensue. Antitumor efficacy (stable or decreased tumor remnant) is reported in >90% of cases (125). AEs include local toxicity such as pituitary deficiencies reported in 20% to 50% of patients and usually correlated with dose to the pituitary stalk (126). Other longer-term AEs may include cognitive dysfunction, increased risk of stroke, and radiation-induced tumors. These AEs have been reported with fractionated radiotherapy, up to 20 years after the procedure (127), and very long-term data with stereotaxy are still lacking. The risks of such AEs are thus uncertain (128). Surprisingly, there is a risk of delayed recurrence in patients considered in remission after stereotaxy: this can be observed 10 to 20 years after the procedure. This recurrence is usually due to a poorly defined target, implying that the dose did not perfectly cover the remnant, or to aggressive adenoma behavior (129). This emphasizes the need for prolonged follow-up to quickly diagnose long-term AEs and recurrences.

Conformal techniques use the principle of fractionation to destroy tumors gradually while allowing normal pituitary cells to recover. Protection for critical surrounding structures is based on the principle of “biological sparing” (ie, that the biochemical properties of normal and adenoma cells are different and respond differently to radiation). Conformal techniques are more frequently used for larger adenoma volumes or for aggressive lesions. Antisecretory therapeutic efficacy is evident in 50% to 70% of patients (127, 129, 130). Proton beam treatment uses the Bragg peak principle: accelerated protons deposit a little dose all along their trajectory and then release a sharp peak at a controllable depth. This technical sparing should theoretically decrease the risk of extra-pituitary AEs. However, very few studies are published on proton beam for acromegaly (131, 132). The precise role, efficacy, and toxicity of proton beam treatment remain to be determined.

The optimal radiation technique depends on the volume of the postoperative remnant, the proximity of structures to be protected, and pre-radiotherapy evaluation (pituitary deficiencies, well-defined remnant). The major caveat is the time to maximal efficacy, requiring drugs to control hypersecretion, and the uncertainty of very long-term side effects on brain structure. Focused radiation techniques still have a role in management and should be considered as second- or third-line treatments.

## Conclusions

Many patients with GH-secreting PA require multimodal therapy with surgery, medications, and, less frequently, radiation. Acromegaly treatments, delayed diagnosis, and persistent comorbidities also contribute to the disease burden. The recently expanded spectrum of acromegaly medications, although not yet available in all countries, offers a more individualized approach after consideration of biochemical, histological, and imaging factors; glycemic status; and patient preference regarding drug administration. Optimal management of acromegaly ideally involves experienced multidisciplinary teams and shared decision-making.

## Data Availability

Data sharing is not applicable to this article as no data sets were generated or analyzed during the present study.

## Abbreviations

Acro-TSQ: Acromegaly Treatment Satisfaction Questionnaire
AE: adverse event
AIP: aryl hydrocarbon receptor interacting-protein
ATG: autogel
BMI: body mass index
D2R: dopamine type 2 receptor
EMA: European Medicines Agency
FDA: US Food and Drug Administration
GH: growth hormone
IGF-I: insulin-like growth factor 1
IHC: immunohistochemistry
IM: intramuscular
LAR: long-acting release
MRI: magnetic resonance imaging
OOC: oral octreotide capsule
PA: pituitary adenoma
QOL: quality of life
SC: subcutaneous
SRL: somatostatin receptor ligand
SSTR: somatostatin receptor subtype

## References

[1] Melmed S, di Filippo L, Fleseriu M, et al Consensus on acromegaly therapeutic outcomes: an update. Nat Rev Endocrinol. 2025;21(11):718‐737. 40804505 10.1038/s41574-025-01148-2

[2] Baussart B, Zoli M, Passeri T, et al Outcome of acromegalic patients undergoing endoscopic endonasal surgery: collaborative French and Italian cohort, a 25-year experience. Neurosurg Rev. 2025;48(1):654. 40965517 10.1007/s10143-025-03797-3

[3] Bollerslev J, Heck A, Olarescu NC. Management of endocrine disease: individualised management of acromegaly. Eur J Endocrinol. 2019;181(2):R57‐R71. 31100716 10.1530/EJE-19-0124

[4] Giustina A, Biermasz N, Casanueva FF, et al Consensus on criteria for acromegaly diagnosis and remission. Pituitary. 2024;27(1):7‐22. 37923946 10.1007/s11102-023-01360-1 PMC10837217

[5] Kinoshita Y, Taguchi A, Yamasaki F, Onishi S, Tominaga A, Horie N. Management policy for postoperative acromegaly patients with normal IGF-1 and high GH levels on oral glucose tests. Pituitary. 2024;28(1):4. 39724486 10.1007/s11102-024-01487-9

[6] Graillon T, Castinetti F, Boucekine M, et al Fluctuation analysis of postoperative secretory status in patients operated for acromegaly. Ann Endocrinol (Paris). 2020;81(1):11‐17. 31982107 10.1016/j.ando.2019.11.002

[7] Starnoni D, Daniel RT, Marino L, Pitteloud N, Levivier M, Messerer M. Surgical treatment of acromegaly according to the 2010 remission criteria: systematic review and meta-analysis. Acta Neurochir (Wien). 2016;158(11):2109‐2121. 27586125 10.1007/s00701-016-2903-4

[8] Agrawal N, Ioachimescu AG. Prognostic factors of biochemical remission after transsphenoidal surgery for acromegaly: a structured review. Pituitary. 2020;23(5):582‐594. 32602066 10.1007/s11102-020-01063-x

[9] Chen C-J, Ironside N, Pomeraniec IJ, et al Microsurgical versus endoscopic transsphenoidal resection for acromegaly: a systematic review of outcomes and complications. Acta Neurochir (Wien). 2017;159(11):2193‐2207. 28913667 10.1007/s00701-017-3318-6 PMC6558977

[10] Oberman DZ, Sanchez-Garavito E, Perez-Vega C, et al Selective resection of the medial wall of the cavernous sinus in pituitary surgery: results of a prospective single center analysis. Pituitary. 2025;28(1):19. 39863739 10.1007/s11102-024-01476-y

[11] Calandrelli R, De Lucia D, Chiloiro S, et al Histopathological and radiological predictors of surgical remission failure in GH-secreting pituitary adenomas. Pituitary. 2026;29(1):39. 41663696 10.1007/s11102-025-01635-9

[12] Almeida JP, Ruiz-Treviño AS, Liang B, et al Reoperation for growth hormone-secreting pituitary adenomas: report on an endonasal endoscopic series with a systematic review and meta-analysis of the literature. J Neurosurg. 2018;129(2):404‐416. 28862548 10.3171/2017.2.JNS162673

[13] Anthony JR, Alwahab UA, Kanakiya NK, et al Significant elevation of growth hormone level impacts surgical outcomes in acromegaly. Endocr Pract. 2015;21(9):1001‐1009. 26121434 10.4158/EP14587.OR

[14] Coopmans EC, Postma MR, Wolters TLC, et al Predictors for remission after transsphenoidal surgery in acromegaly: a Dutch multicenter study. J Clin Endocrinol Metab. 2021;106(6):1783‐1792. 33544833 10.1210/clinem/dgab069 PMC8118364

[15] Del Corso LM, Mesa Junior CO, Andrade VFC, Fidalski SZK, Boguszewski CL. Diagnostic, therapeutic, and prognostic characteristics of patients with acromegaly according to tumor size at diagnosis. Pituitary. 2024;27(5):537‐544. 39088137 10.1007/s11102-024-01432-w

[16] Konar S, Yeole U, Shukla D, Bhat DI, Sadashiva N, Devi BI. Predictors of remission of acromegaly following surgical treatment in growth hormone-secreting pituitary adenoma. J Neurol Surg B Skull Base. 2024;85(3):261‐266. 38721370 10.1055/s-0043-57233 PMC11076092

[17] Cardinal T, Collet C, Wedemeyer M, et al Postoperative GH and degree of reduction in IGF-1 predicts postoperative hormonal remission in acromegaly. Front Endocrinol (Lausanne). 2021;12:743052. 34867787 10.3389/fendo.2021.743052 PMC8637049

[18] Freda PU, Wardlaw SL, Post KD. Long-term endocrinological follow-up evaluation in 115 patients who underwent transsphenoidal surgery for acromegaly. J Neurosurg. 1998;89(3):353‐358. 9724106 10.3171/jns.1998.89.3.0353

[19] Guo X, Zhang R, Zhang D, et al Determinants of immediate and long-term remission after initial transsphenoidal surgery for acromegaly and outcome patterns during follow-up: a longitudinal study on 659 patients. J Neurosurg. 2022;137(3):618‐628. 35171834 10.3171/2021.11.JNS212137

[20] Jane JA Jr, Starke RM, Elzoghby MA, et al Endoscopic transsphenoidal surgery for acromegaly: remission using modern criteria, complications, and predictors of outcome. J Clin Endocrinol Metab. 2011;96(9):2732‐2740. 21715544 10.1210/jc.2011-0554

[21] Kraljevic I, Solak M, Kovac D, et al Early postoperative growth hormone measurement as a predictive marker for acromegaly remission. J Neuroendocrinol. 2024;36(11):e13434. 39056158 10.1111/jne.13434

[22] Krieger MD, Couldwell WT, Weiss MH. Assessment of long-term remission of acromegaly following surgery. J Neurosurg. 2003;98(4):719‐724. 12691394 10.3171/jns.2003.98.4.0719

[23] Chong L, Lou Y, Chen X, et al Comparison of the clinical and prognostic characteristics of patients with different pathological types in acromegaly. Front Endocrinol (Lausanne). 2025;16:1571598. 40421250 10.3389/fendo.2025.1571598 PMC12104045

[24] Araujo-Castro M, Biagetti B, Menéndez Torre E, et al Differences between GH- and PRL-cosecreting and GH-secreting pituitary adenomas: a series of 604 cases. J Clin Endocrinol Metab. 2024;109(12):e2178‐e2187. 38436926 10.1210/clinem/dgae126

[25] Cremaschi A, Sala E, Lavezzi E, et al Recurrence in acromegaly: two tertiary centers experience and review of the literature. J Endocrinol Invest. 2024;47(9):2269‐2277. 38502285 10.1007/s40618-024-02321-6 PMC11368993

[26] Colao A, Grasso LFS, Giustina A, et al Acromegaly. Nat Rev Dis Primers. 2019;5(1):20. 30899019 10.1038/s41572-019-0071-6

[27] Gadelha MR, Wildemberg LE, Marques NV, Kasuki L. Medical treatment of acromegaly: navigating the present, shaping the future. Endocr Rev. 2025;46(6):838‐855. 40644375 10.1210/endrev/bnaf020

[28] Melmed S, Bronstein MD, Chanson P, et al A consensus statement on acromegaly therapeutic outcomes. Nat Rev Endocrinol. 2018;14(9):552‐561. 30050156 10.1038/s41574-018-0058-5 PMC7136157

[29] Colao A, Auriemma RS, Rebora A, et al Significant tumour shrinkage after 12 months of lanreotide autogel-120 mg treatment given first-line in acromegaly. Clin Endocrinol (Oxf). 2009;71(2):237‐245. 19094074 10.1111/j.1365-2265.2008.03503.x

[30] Melmed S, Cook D, Schopohl J, Goth MI, Lam KS, Marek J. Rapid and sustained reduction of serum growth hormone and insulin-like growth factor-1 in patients with acromegaly receiving lanreotide autogel therapy: a randomized, placebo-controlled, multicenter study with a 52 week open extension. Pituitary. 2010;13(1):18‐28. 19639415 10.1007/s11102-009-0191-1 PMC2807598

[31] Fleseriu M, Barkan A, Brue T, et al Treatment patterns, adherence, persistence, and health care resource utilization in acromegaly: a real-world analysis. J Endocr Soc. 2023;7(10):bvad104. 37705695 10.1210/jendso/bvad104 PMC10496868

[32] Chiloiro S, Giampietro A, Giambo P, et al IGF-I levels during standard lanreotide dose predicts biochemical outcome of high-frequency regimen in acromegaly. Pituitary. 2024;28(1):7. 39724447 10.1007/s11102-024-01479-9

[33] Fleseriu M, Zhang Z, Hanman K, et al A systematic literature review to evaluate extended dosing intervals in the pharmacological management of acromegaly. Pituitary. 2023;26(1):9‐41. 36447058 10.1007/s11102-022-01285-1 PMC9708130

[34] Coopmans EC, Korevaar TIM, van Meyel SWF, et al Multivariable prediction model for biochemical response to first-generation somatostatin receptor ligands in acromegaly. J Clin Endocrinol Metab. 2020;105(9):2964‐2974. 10.1210/clinem/dgaa387 32589751

[35] Ilie MD, Tabarin A, Vasiljevic A, et al Predictive factors of somatostatin receptor ligand response in acromegaly—a prospective study. J Clin Endocrinol Metab. 2022;107(11):2982‐2991. 36136828 10.1210/clinem/dgac512

[36] Chiloiro S, Moroni R, Giampietro A, et al The multibiomarker acro-TIME score predicts fg-SRLs response: preliminary results of a retrospective acromegaly cohort. J Clin Endocrinol Metab. 2024;109(5):1341‐1350. 37975821 10.1210/clinem/dgad673

[37] Wildemberg LE, da Silva Camacho AH, Miranda RL, et al Machine learning-based prediction model for treatment of acromegaly with first-generation somatostatin receptor ligands. J Clin Endocrinol Metab. 2021;106(7):2047‐2056. 33686418 10.1210/clinem/dgab125

[38] Gil J, Marques-Pamies M, Sampedro M, et al Data mining analyses for precision medicine in acromegaly: a proof of concept. Sci Rep. 2022;12(1):8979. 35643771 10.1038/s41598-022-12955-2 PMC9148300

[39] Lin W, Shi S, Zheng Y, Laws E, Smith T, Min L. Integrative machine learning approach for predicting resistance to first-generation receptor ligands in acromegaly. J Clin Endocrinol Metab. 2026;111(2):484‐496. 40569033 10.1210/clinem/dgaf375

[40] Marques-Pamies M, Gil J, Sampedro-Nunez M, et al Personalized medicine in acromegaly: the ACROFAST study. J Clin Endocrinol Metab. 2024;110(1):30‐40. 38943661 10.1210/clinem/dgae444 PMC11651705

[41] Caron PJ, Bevan JS, Petersenn S, et al Tumor shrinkage with lanreotide autogel 120 mg as primary therapy in acromegaly: results of a prospective multicenter clinical trial. J Clin Endocrinol Metab. 2014;99(4):1282‐1290. 24423301 10.1210/jc.2013-3318 PMC4009579

[42] Benderradji H, Vernotte E, Soto Ares G, et al Efficacy of lanreotide 120 mg primary therapy on tumour shrinkage and ophthalmologic symptoms in acromegaly after 1 month. Clin Endocrinol (Oxf). 2022;97(1):52‐63. 35470446 10.1111/cen.14748

[43] Bevan JS, Atkin SL, Atkinson AB, et al Primary medical therapy for acromegaly: an open, prospective, multicenter study of the effects of subcutaneous and intramuscular slow-release octreotide on growth hormone, insulin-like growth factor-I, and tumor size. J Clin Endocrinol Metab. 2002;87(10):4554‐4563. 12364434 10.1210/jc.2001-012012

[44] Marques P, Barry S, Carlsen E, et al Pituitary tumour fibroblast-derived cytokines influence tumour aggressiveness. Endocr Relat Cancer. 2019;26(12):853‐865. 31645017 10.1530/ERC-19-0327

[45] Chiloiro S, De Marinis L. The immune microenviroment in somatotropinomas: from biology to personalized and target therapy. Rev Endocr Metab Disord. 2023;24(2):283‐295. 36658300 10.1007/s11154-022-09782-1 PMC10023617

[46] Albarel F, Cuny T, Graillon T, Dufour H, Brue T, Castinetti F. Preoperative medical treatment for patients with acromegaly: yes or no? J Endocr Soc. 2022;6(9):bvac114. 35965944 10.1210/jendso/bvac114 PMC9368018

[47] Yang C, Li G, Jiang S, Bao X, Wang R. Preoperative somatostatin analogues in patients with newly-diagnosed acromegaly: a systematic review and meta-analysis of comparative studies. Sci Rep. 2019;9(1):14070. 31575930 10.1038/s41598-019-50639-6 PMC6773739

[48] Ye W, Liu Z. Real-world drug safety study of somatostatin analogs based on the food and drug administration adverse event reporting system database. Eur J Pharmacol. 2025;1005:178053. 40840589 10.1016/j.ejphar.2025.178053

[49] Geer EB, Sisco J, Adelman DT, et al Patient reported outcome data from acromegaly patients treated with injectable somatostatin receptor ligands (SRLs) in routine clinical practice. BMC Endocr Disord. 2020;20(1):117. 32736547 10.1186/s12902-020-00595-4 PMC7393879

[50] Strasburger CJ, Karavitaki N, Störmann S, et al Patient-reported outcomes of parenteral somatostatin analogue injections in 195 patients with acromegaly. Eur J Endocrinol. 2016;174(3):355‐362. 26744896 10.1530/EJE-15-1042 PMC4722610

[51] Boguszewski CL, Korbonits M, Artignan A, et al Evaluating home injection compared with healthcare-setting injection of somatostatin analogs: a systematic literature review. Endocrine. 2023;79(3):527‐536. 36369434 10.1007/s12020-022-03227-0 PMC9651885

[52] Akirov A, Masri-Iraqi H, Gorshtein A, Duskin-Bitan H, Kaminer K, Shimon I. Benefits of a nurse-led home injection service for acromegaly patients treated with somatuline autogel. Endocrine. 2021;71(2):453‐458. 33098539 10.1007/s12020-020-02529-5

[53] Cella D, Evans J, Feuilly M, et al Patient and healthcare provider perspectives of first-generation somatostatin analogs in the management of neuroendocrine tumors and acromegaly: a systematic literature review. Adv Ther. 2021;38(2):969‐993. 33432541 10.1007/s12325-020-01600-x PMC7799425

[54] Fleseriu M, Molitch M, Dreval A, et al Disease and treatment-related burden in patients with acromegaly who are biochemically controlled on injectable somatostatin receptor ligands. Front Endocrinol (Lausanne). 2021;12:627711. 33790860 10.3389/fendo.2021.627711 PMC8006928

[55] Remba-Shapiro I, Schweizer JROL, Moscona-Nissan A, et al Insulin-like growth factor-I and symptoms of acromegaly according to time since somatostatin receptor ligand injection. Eur J Endocrinol. 2025;193(4):564‐573. 41001687 10.1093/ejendo/lvaf200

[56] Brayden DJ, Maher S. Transient permeation enhancer® (TPE®) technology for oral delivery of octreotide: a technological evaluation. Expert Opin Drug Deliv. 2021;18(10):1501‐1512. 34128734 10.1080/17425247.2021.1942838

[57] Labadzhyan A, Nachtigall LB, Fleseriu M, et al Oral octreotide capsules for the treatment of acromegaly: comparison of 2 phase 3 trial results. Pituitary. 2021;24(6):943‐953. 34173129 10.1007/s11102-021-01163-2 PMC8550586

[58] Samson SL, Nachtigall LB, Fleseriu M, et al Maintenance of acromegaly control in patients switching from injectable somatostatin receptor ligands to oral octreotide. J Clin Endocrinol Metab. 2020;105(10):e3785‐e3797. 32882036 10.1210/clinem/dgaa526 PMC7470473

[59] Samson SL, Nachtigall LB, Fleseriu M, et al Durable biochemical response and safety with oral octreotide capsules in acromegaly. Eur J Endocrinol. 2022;187(6):733‐741. 36173649 10.1530/EJE-22-0220 PMC9641789

[60] Fleseriu M, Dreval A, Bondar I, et al Maintenance of response to oral octreotide compared with injectable somatostatin receptor ligands in patients with acromegaly: a phase 3, multicentre, randomised controlled trial. Lancet Diabetes Endocrinol. 2022;10(2):102‐111. 34953531 10.1016/S2213-8587(21)00296-5

[61] Fleseriu M, Molitch M, Dreval A, et al MPOWERED trial open-label extension: long-term efficacy and safety data for oral octreotide capsules in acromegaly. J Clin Endocrinol Metab. 2023;108(12):3214‐3222. 37319438 10.1210/clinem/dgad365 PMC10655542

[62] Fleseriu M, Nachtigall LB, Samson SL, Melmed S. Oral octreotide capsules for acromegaly treatment: application of clinical trial insights to real-world use. Expert Rev Endocrinol Metab. 2024;19(4):367‐375. 38842362 10.1080/17446651.2024.2363540

[63] Ferone D, Freda P, Katznelson L, et al Octreotide subcutaneous depot for acromegaly: a randomized, double-blind, placebo-controlled phase 3 trial, ACROINNOVA 1. J Clin Endocrinol Metab. 2025;110(6):1729‐1739. 39378125 10.1210/clinem/dgae707 PMC12086390

[64] Spencer-Segal JL, Silverstein JM, Gillis-Janusewska A, et al SUN-051 CAM2029, an octreotide subcutaneous depot, provides long-term and sustained biochemical control of acromegaly: final analysis of the core part of the ACROINNOVA 2 phase 3 trial. J Endocr Soc. 2025;9(Supplement_1):A863.

[65] Madan A, Markison S, Betz SF, et al Paltusotine, a novel oral once-daily nonpeptide SST2 receptor agonist, suppresses GH and IGF-1 in healthy volunteers. Pituitary. 2022;25(2):328‐339. 35000098 10.1007/s11102-021-01201-z PMC8894159

[66] Cristante J, Castinetti F. New treatments for acromegaly: is a revolution underway? Ann Endocrinol (Paris). 2025;86(2):101710. 39961483 10.1016/j.ando.2025.101710

[67] Gadelha MR, Casagrande A, Strasburger CJ, et al Acromegaly disease control maintained after switching from injected somatostatin receptor ligands to oral paltusotine. J Clin Endocrinol Metab. 2024;110(1):228‐237. 38828555 10.1210/clinem/dgae385 PMC11651685

[68] Biller BM, Casagrande A, Elenkova A, et al 12535 efficacy and safety of once-daily oral paltusotine in medically untreated patients with acromegaly: results from the phase 3, randomized, placebo-controlled PATHFNDR-2 study. J Endocr Soc. 2024;8(Suppl_1):A625.

[69] Castinetti F, Brue T. Paltusotine: a step toward precision medicine in acromegaly. J Clin Endocrinol Metab. 2025;110(3):e897‐e898. 39004834 10.1210/clinem/dgae489

[70] Colao A, Bronstein MD, Freda P, et al Pasireotide versus octreotide in acromegaly: a head-to-head superiority study. J Clin Endocrinol Metab. 2014;99(3):791‐799. 24423324 10.1210/jc.2013-2480 PMC3965714

[71] Gadelha MR, Bronstein MD, Brue T, et al Pasireotide versus continued treatment with octreotide or lanreotide in patients with inadequately controlled acromegaly (PAOLA): a randomised, phase 3 trial. Lancet Diabetes Endocrinol. 2014;2(11):875‐884. 25260838 10.1016/S2213-8587(14)70169-X

[72] Colao A, Bronstein MD, Brue T, et al Pasireotide for acromegaly: long-term outcomes from an extension to the phase III PAOLA study. Eur J Endocrinol. 2020;182(6):583. 32217809 10.1530/EJE-19-0762 PMC7222286

[73] Muhammad A, van der Lely AJ, Delhanty PJD, et al Efficacy and safety of switching to pasireotide in patients with acromegaly controlled with pegvisomant and first-generation somatostatin analogues (PAPE study). J Clin Endocrinol Metab. 2018;103(2):586‐595. 29155991 10.1210/jc.2017-02017

[74] Mondin A, Manara R, Voltan G, et al Pasireotide-induced shrinkage in GH and ACTH secreting pituitary adenoma: a systematic review and meta-analysis. Front Endocrinol (Lausanne). 2022;13:935759. 35846311 10.3389/fendo.2022.935759 PMC9283714

[75] Biagetti B, Araujo-Castro M, Tebe C, Marazuela M, Puig-Domingo M. Real-world evidence of effectiveness and safety of pasireotide in the treatment of acromegaly: a systematic review and meta-analysis. Rev Endocr Metab Disord. 2025;26(1):97‐111. 39527181 10.1007/s11154-024-09928-3 PMC11790789

[76] Pirchio R, Auriemma RS, Vergura A, Pivonello R, Colao A. Long-term pasireotide therapy in acromegaly: extensive real-life experience of a referral center. J Endocrinol Invest. 2024;47(8):1887‐1901. 38532073 10.1007/s40618-023-02299-7 PMC11266387

[77] Coopmans EC, Schneiders JJ, El-Sayed N, et al T2-signal intensity, SSTR expression, and somatostatin analogs efficacy predict response to pasireotide in acromegaly. Eur J Endocrinol. 2020;182(6):595‐605. 32375119 10.1530/EJE-19-0840

[78] Gadelha MR, Gu F, Bronstein MD, et al Risk factors and management of pasireotide-associated hyperglycemia in acromegaly. Endocr Connect. 2020;9(12):1178‐1190. 33434154 10.1530/EC-20-0361 PMC7774766

[79] Störmann S, Meyhöfer SM, Groener JB, et al Management of pasireotide-induced hyperglycemia in patients with acromegaly: an experts’ consensus statement. Front Endocrinol (Lausanne). 2024;15:1348990. 38405148 10.3389/fendo.2024.1348990 PMC10884330

[80] Giustina A, Barkhoudarian G, Beckers A, et al Multidisciplinary management of acromegaly: a consensus. Rev Endocr Metab Disord. 2020;21(4):667‐678. 32914330 10.1007/s11154-020-09588-z PMC7942783

[81] Fleseriu M, Biller BMK, Freda PU, et al A pituitary society update to acromegaly management guidelines. Pituitary. 2021;24(1):1‐13. 33079318 10.1007/s11102-020-01091-7 PMC7864830

[82] Brue T, Rahabi H, Barry A, et al Position statement on the diagnosis and management of acromegaly: the French National Diagnosis and Treatment Protocol (NDTP). Ann Endocrinol (Paris). 2023;84(6):697‐710. 37579837 10.1016/j.ando.2023.08.003

[83] Strasburger CJ, Mattsson A, Wilton P, Aydin F, Hey-Hadavi J, Biller BMK. Increasing frequency of combination medical therapy in the treatment of acromegaly with the GH receptor antagonist pegvisomant. Eur J Endocrinol. 2018;178(4):321‐329. 29371335 10.1530/EJE-17-0996 PMC5863474

[84] Trainer PJ, Drake WM, Katznelson L, et al Treatment of acromegaly with the growth hormone-receptor antagonist pegvisomant. N Engl J Med. 2000;342(16):1171‐1177. 10770982 10.1056/NEJM200004203421604

[85] van der Lely AJ, Hutson RK, Trainer PJ, et al Long-term treatment of acromegaly with pegvisomant, a growth hormone receptor antagonist. Lancet. 2001;358(9295):1754‐1759. 11734231 10.1016/s0140-6736(01)06844-1

[86] Giampietro A, Chiloiro S, Urbani C, et al Factors associated with disease control failure in acromegaly patients treated with pegvisomant: an ACROSTUDY analysis. Endocr Connect. 2024;13(3):e230247. 38197875 10.1530/EC-23-0247 PMC10895310

[87] Franck SE, Korevaar TIM, Petrossians P, et al A multivariable prediction model for pegvisomant dosing: monotherapy and in combination with long-acting somatostatin analogues. Eur J Endocrinol. 2017;176(4):421‐431. 28100630 10.1530/EJE-16-0956

[88] Parkinson C, Burman P, Messig M, Trainer PJ. Gender, body weight, disease activity, and previous radiotherapy influence the response to pegvisomant. J Clin Endocrinol Metab. 2007;92(1):190‐195. 17077131 10.1210/jc.2006-1412

[89] Besser GM, Burman P, Daly AF. Predictors and rates of treatment-resistant tumor growth in acromegaly. Eur J Endocrinol. 2005;153(2):187‐193. 16061822 10.1530/eje.1.01968

[90] Jimenez C, Burman P, Abs R, et al Follow-up of pituitary tumor volume in patients with acromegaly treated with pegvisomant in clinical trials. Eur J Endocrinol. 2008;159(5):517‐523. 18708436 10.1530/EJE-08-0205

[91] Leonart LP, Tonin FS, Ferreira VL, Fernandez-Llimos F, Pontarolo R. Effectiveness and safety of pegvisomant: a systematic review and meta-analysis of observational longitudinal studies. Endocrine. 2019;63(1):18‐26. 30145746 10.1007/s12020-018-1729-7

[92] Buchfelder M, van der Lely AJ, Biller BMK, et al Long-term treatment with pegvisomant: observations from 2090 acromegaly patients in acrostudy. Eur J Endocrinol. 2018;179(6):419‐427. 30325178 10.1530/EJE-18-0616

[93] Brue T, Lindberg A, Jan van der Lely A, et al Diabetes in patients with acromegaly treated with pegvisomant: observations from acrostudy. Endocrine. 2019;63(3):563‐572. 30474822 10.1007/s12020-018-1792-0 PMC6420440

[94] Moller N, Jorgensen JO. Effects of growth hormone on glucose, lipid, and protein metabolism in human subjects. Endocr Rev. 2009;30(2):152‐177. 19240267 10.1210/er.2008-0027

[95] Gatto F, Arecco A, Amarù J, et al Differential impact of medical therapies for acromegaly on glucose metabolism. Int J Mol Sci. 2025;26(2):465. 39859181 10.3390/ijms26020465 PMC11764544

[96] Barkan AL, Burman P, Clemmons DR, et al Glucose homeostasis and safety in patients with acromegaly converted from long-acting octreotide to pegvisomant. J Clin Endocrinol Metab. 2005;90(10):5684‐5691. 16076947 10.1210/jc.2005-0331

[97] Salvatori R, Maffei P, Webb SM, et al Patient-reported outcomes in patients with acromegaly treated with pegvisomant in the ACROSTUDY extension: a real-world experience. Pituitary. 2022;25(3):420‐432. 35022929 10.1007/s11102-022-01206-2

[98] Dichtel LE, Kimball A, Yuen KCJ, et al Effects of growth hormone receptor antagonism and somatostatin analog administration on quality of life in acromegaly. Clin Endocrinol (Oxf). 2021;94(1):58‐65. 32779234 10.1111/cen.14309 PMC9217182

[99] Neggers SJCMM, de Herder WW, Feelders RA, van der Lely AJ. Conversion of daily pegvisomant to weekly pegvisomant combined with long-acting somatostatin analogs, in controlled acromegaly patients. Pituitary. 2011;14(3):253‐258. 21221818 10.1007/s11102-010-0289-5 PMC3146981

[100] Muhammad A, van der Lely AJ, O'Connor RD, et al What is the efficacy of switching to weekly pegvisomant in acromegaly patients well controlled on combination therapy? Eur J Endocrinol. 2016;174(5):663‐667. 26903550 10.1530/EJE-15-1150

[101] Camara R, Venegas E, Garcia-Arnes JA, et al Treatment adherence to pegvisomant in patients with acromegaly in Spain: PEGASO study. Pituitary. 2019;22(2):137‐145. 30756345 10.1007/s11102-019-00943-1

[102] Feenstra J, de Herder WW, ten Have SM, et al Combined therapy with somatostatin analogues and weekly pegvisomant in active acromegaly. Lancet. 2005;365(9471):1644‐1646. 15885297 10.1016/S0140-6736(05)63011-5

[103] Trainer PJ, Ezzat S, D'Souza GA, Layton G, Strasburger CJ. A randomized, controlled, multicentre trial comparing pegvisomant alone with combination therapy of pegvisomant and long-acting octreotide in patients with acromegaly. Clin Endocrinol (Oxf). 2009;71(4):549‐557. 19438906 10.1111/j.1365-2265.2009.03620.x

[104] Neggers SJCMM, Franck SE, de Rooij FWM, et al Long-term efficacy and safety of pegvisomant in combination with long-acting somatostatin analogs in acromegaly. J Clin Endocrinol Metab. 2014;99(10):3644‐3652. 24937542 10.1210/jc.2014-2032

[105] Ma L, Luo D, Yang T, et al Combined therapy of somatostatin analogues with pegvisomant for the treatment of acromegaly: a meta-analysis of prospective studies. BMC Endocr Disord. 2020;20(1):126. 32811475 10.1186/s12902-020-0545-2 PMC7433060

[106] Jørgensen JOL, Feldt-Rasmussen U, Frystyk J, et al Cotreatment of acromegaly with a somatostatin analog and a growth hormone receptor antagonist. J Clin Endocrinol Metab. 2005;90(10):5627‐5631. 16046586 10.1210/jc.2005-0531

[107] van der Lely AJ, Muller A, Janssen JA, et al Control of tumor size and disease activity during cotreatment with octreotide and the growth hormone receptor antagonist pegvisomant in an acromegalic patient. J Clin Endocrinol Metab. 2001;86(2):478‐481. 11157994 10.1210/jcem.86.2.7206

[108] Leung KC, Doyle N, Ballesteros M, Waters MJ, Ho KK. Insulin regulation of human hepatic growth hormone receptors: divergent effects on biosynthesis and surface translocation. J Clin Endocrinol Metab. 2000;85(12):4712‐4720. 11134133 10.1210/jcem.85.12.7017

[109] Neggers SJCMM, van Aken MO, de Herder WW, et al Quality of life in acromegalic patients during long-term somatostatin analog treatment with and without pegvisomant. J Clin Endocrinol Metab. 2008;93(10):3853‐3859. 18647806 10.1210/jc.2008-0669

[110] De Marinis L, Bianchi A, Fusco A, et al Long-term effects of the combination of pegvisomant with somatostatin analogs (SSA) on glucose homeostasis in non-diabetic patients with active acromegaly partially resistant to SSA. Pituitary. 2007;10(3):227‐232. 17484056 10.1007/s11102-007-0037-7

[111] Bonert V, Mirocha J, Carmichael J, Yuen KCJ, Araki T, Melmed S. Cost-effectiveness and efficacy of a novel combination regimen in acromegaly: a prospective, randomized trial. J Clin Endocrinol Metab. 2020;105(9):e3236‐e3245. 10.1210/clinem/dgaa444 32754748

[112] Coopmans EC, van Meyel SWF, van der Lely AJ, Neggers SJCMM. The position of combined medical treatment in acromegaly. Arch Endocrinol Metab. 2019;63(6):646‐652. 31939490 10.20945/2359-3997000000195 PMC10522231

[113] Chiloiro S, Bima C, Tartaglione T, et al Pasireotide and pegvisomant combination treatment in acromegaly resistant to second-line therapies: a longitudinal study. J Clin Endocrinol Metab. 2019;104(11):5478‐5482. 31219586 10.1210/jc.2019-00825

[114] Chiloiro S, Giampietro A, Mirra F, et al Pegvisomant and pasireotide LAR as second line therapy in acromegaly: clinical effectiveness and predictors of response. Eur J Endocrinol. 2021;184(2):217‐229. 33136550 10.1530/EJE-20-0767

[115] Giraldi EA, Ioachimescu AG. The role of dopamine agonists in pituitary adenomas. Endocrinol Metab Clin North Am. 2020;49(3):453‐474. 32741482 10.1016/j.ecl.2020.05.006

[116] Shimon I . Real-world value of cabergoline in the treatment of acromegaly. Best Pract Res Clin Endocrinol Metab. 2024;38(4):101887. 38443225 10.1016/j.beem.2024.101887

[117] Higham CE, Atkinson AB, Aylwin S, et al Effective combination treatment with cabergoline and low-dose pegvisomant in active acromegaly: a prospective clinical trial. J Clin Endocrinol Metab. 2012;97(4):1187‐1193. 22278424 10.1210/jc.2011-2603

[118] Sandret L, Maison P, Chanson P. Place of cabergoline in acromegaly: a meta-analysis. J Clin Endocrinol Metab. 2011;96(5):1327‐1335. 21325455 10.1210/jc.2010-2443

[119] Bernabeu I, Alvarez-Escolá C, Paniagua AE, et al Pegvisomant and cabergoline combination therapy in acromegaly. Pituitary. 2013;16(1):101‐108. 22396133 10.1007/s11102-012-0382-z

[120] Fleseriu M, Dreval AV, Pokramovich Y, et al Addition of cabergoline to oral octreotide capsules may improve biochemical control in patients with acromegaly who are inadequately controlled with monotherapy. Endocr Abs. 2021;73:PEP8.3.

[121] Amodru V, Sahakian N, Piazzola C, et al Changes in multi-modality management of acromegaly in a tertiary centre over 2 decades. Pituitary. 2024;27(3):294‐302. 38521837 10.1007/s11102-024-01387-y

[122] Maione L, Brue T, Beckers A, et al Changes in the management and comorbidities of acromegaly over three decades: the French acromegaly registry. Eur J Endocrinol. 2017;176(5):645‐655. 28246150 10.1530/EJE-16-1064

[123] Castinetti F, Régis J, Dufour H, Brue T. Role of stereotactic radiosurgery in the management of pituitary adenomas. Nat Rev Endocrinol. 2010;6(4):214‐223. 20177403 10.1038/nrendo.2010.4

[124] Singh R, Didwania P, Lehrer EJ, et al Stereotactic radiosurgery for acromegaly: an international systematic review and meta-analysis of clinical outcomes. J Neurooncol. 2020;148(3):401‐418. 32506372 10.1007/s11060-020-03552-2

[125] Ding D, Mehta GU, Patibandla MR, et al Stereotactic radiosurgery for acromegaly: an international multicenter retrospective cohort study. Neurosurgery. 2019;84(3):717‐725. 29757421 10.1093/neuros/nyy178 PMC6505445

[126] Cordeiro D, Xu Z, Mehta GU, et al Hypopituitarism after gamma knife radiosurgery for pituitary adenomas: a multicenter, international study. J Neurosurg. 2019;131(4):1188‐1196. 31369225 10.3171/2018.5.JNS18509 PMC9535685

[127] Hamblin R, Vardon A, Akpalu J, et al Risk of second brain tumour after radiotherapy for pituitary adenoma or craniopharyngioma: a retrospective, multicentre, cohort study of 3679 patients with long-term imaging surveillance. Lancet Diabetes Endocrinol. 2022;10(8):581‐588. 35780804 10.1016/S2213-8587(22)00160-7

[128] Castinetti F, Caron P, Raingeard I, et al Lack of delayed neurocognitive side effects of gamma knife radiosurgery in acromegaly: the later-ac study. Eur J Endocrinol. 2021;186(1):37‐44. 34714763 10.1530/EJE-21-0826

[129] Mantziaris G, Trifiletti DM, Pikis S, Sheehan JP. Stereotactic radiosurgery and fractionated radiation therapy in the management of pituitary tumors. Neurooncol Adv. 2025;7(Suppl 1):i58‐i68. 40718388 10.1093/noajnl/vdae010 PMC12288128

[130] Gonzales-Virla B, Vargas-Ortega G, Martinez-Vazquez KB, et al Efficacy and safety of fractionated conformal radiation therapy in acromegaly: a long-term follow-up study. Endocrine. 2019;65(2):386‐392. 31098940 10.1007/s12020-019-01955-4

[131] Petit JH, Biller BM, Coen JJ, et al Proton stereotactic radiosurgery in management of persistent acromegaly. Endocr Pract. 2007;13(7):726‐734. 18194929 10.4158/EP.13.7.726

[132] Wattson DA, Tanguturi SK, Spiegel DY, et al Outcomes of proton therapy for patients with functional pituitary adenomas. Int J Radiat Oncol Biol Phys. 2014;90(3):532‐539. 25194666 10.1016/j.ijrobp.2014.06.068

